# Intracellular membranes of bacterial endospores are reservoirs for spore core membrane expansion during spore germination

**DOI:** 10.1038/s41598-018-29879-5

**Published:** 2018-07-30

**Authors:** Michael Laue, Hong-Mei Han, Christin Dittmann, Peter Setlow

**Affiliations:** 1 Advanced Light and Electron Microscopy (ZBS 4), Robert Koch Institute, Seestrasse 10, D-13353 Berlin, Germany; 2 Department of Systemic Cell Biology, Max-Planck-Institute of Molecular Physiology, Otto-Hahn-Strasse 11, D-44227 Dortmund, Germany; 3 Molecular Biology and Biophysics, UConn Health, Farmington, CT 06030-3305 USA

## Abstract

Bacterial endospores are formed by certain bacteria, such as *Bacillus subtilis* or the pathogenic *Bacillus anthracis* and *Clostridioides difficile*, to allow survival in environmental conditions which are lethal to vegetative bacteria. The spores possess a particular architecture and molecular inventory which endow them with a remarkable resistance against desiccation, heat and radiation. Another remarkable spore feature is their rapid return to vegetative growth during spore germination and outgrowth. The underlying processes of this latter physiological and morphological transformation involve a number of different events, some of which are mechanistically not entirely understood. One of these events is the expansion of the central spore core, which contains the DNA, RNA and most spore enzymes. To date, it has been unclear how the ~1.3- to 1.6-fold expansion of the core membrane surface area that accompanies core expansion takes place, since this occurs in the absence of significant if any ATP synthesis. In the current work, we demonstrate the presence of intracellular membrane structures in spores located just below the core membrane. During spore germination these internal core membranes disappear when the core size increases, suggesting that they are integrated into the core membrane to allow core expansion. These intracellular membranes are most probably present as more or less compressed vesicles or tubules within the dormant spore core. Investigations of spores from different species suggest that these intracellular membrane structures below the core membrane are a general feature of endospore forming bacteria.

## Introduction

**Figure 1:**
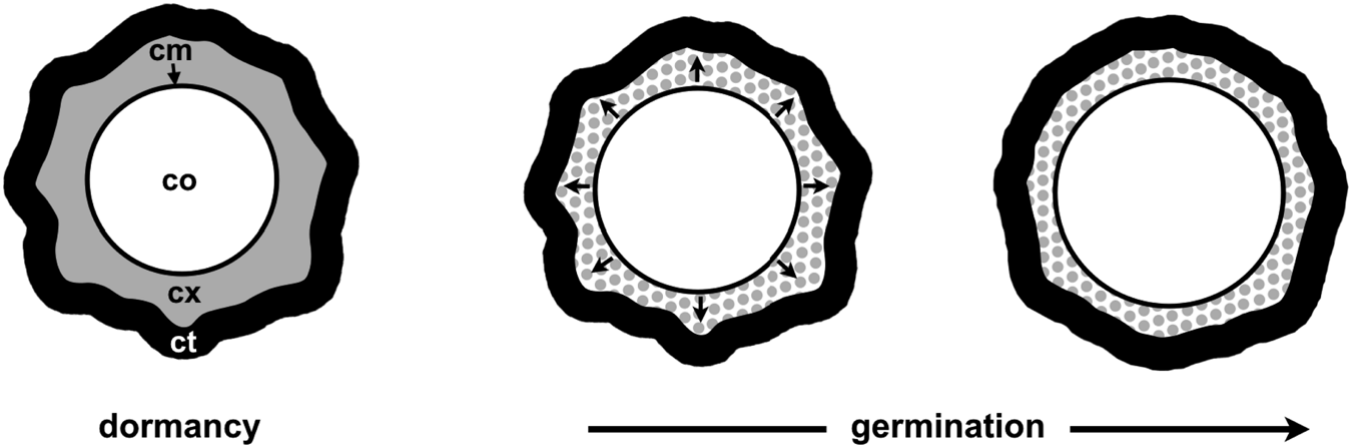
Schematic representation of the morphology of the dormant and germinating spore. The spore contains a core (co) which is limited by a biomembrane (cm) and is surrounded by a cortex (cx) and a multi-layered coat (ct). During germination the cortex is degraded (indicated by stippling) and the core is expanding (arrows) due to water uptake. So far, it is unclear how the membrane is able to accomplish a surface area enlargement to allow the ~2-fold core volume expansion^7^.

Bacteria of the classes Bacilli and Clostridia form endospores to survive harsh environmental conditions such as lack of nutrients^1^. In pathogenic species, like *Bacillus anthracis* or *Clostridioides difficile*, the spore stage often plays a major role in transmission, infection or outbreak of the associated disease^2^. The novel architecture and molecular composition of the spore provides resistance against extreme stresses (e.g. radiation, desiccation, heat)^3^. A multi-layered coat and a cortex layer surround the membrane-bound core^4^ (Fig. 1), which contains spore DNA, RNA and most spore enzymes^3^. During sporulation an outer membrane is formed and localized between the developing cortex and coat, but it is unclear if it persists in the mature spore^1^. Unique features of the central core and the core membrane (CM) include: i) a low water content, as low as 25% of wet weight for spores in water; ii) high core levels, ~25% of core dry weight, of a 1:1 chelate of Ca^2+^ and dipicolinic acid (CaDPA); iii) DNA stabilisation by novel spore-specific proteins; and iv) a relatively impermeable CM. These features are all major reasons for spore’s dormancy and extreme resistance^3^. Notably, under suitable environmental conditions, primarily the presence of nutrients, spores germinate and then outgrow into vegetative bacteria which are capable of reproduction^5^. The germination process is characterized by a sequence of events, including release of all CaDPA and uptake of some core water, and then hydrolysis of spore’s large peptidoglycan cortex, which leads to the uptake of more water into the core (to ~80% of wet weight) and finally to core expansion^5,6^ (Fig. 1). While water uptake is most likely a passive process and cortex hydrolysis is driven by enzymes, it is unclear how the CM is able to increase its surface area approximately 1.3- to 1.6-fold^7^, since lipid biosynthesis is most likely non-existent at this period in germination due to the absence of ATP^5^. One idea to explain the increase in the CM surface area after cortex degradation and water uptake into the core during germination is that the CM relaxes from a compressed state which is generated during sporulation by formation of the cortex^7^. However, the CM of dormant spores reveals no infoldings or significant undulation (see images from cryo-electron microscopy of spores^8,9^) which would indicate such a compressed state and without them the CM would not be able to expand its surface area by 30 to 60%, because model membranes are only able to accomplish an increase of about 2% without losing membrane integrity^10^. An alternative explanation is that the spore has an additional source of membrane, besides the CM, which is inserted into the core membrane during germination to allow the CM surface to increase, but until now, additional sources of membrane could not be found in the core of spores. In the work presented in this paper we show that pre-formed membrane-structures are indeed present within the core of dormant spores just below the CM, and we provide evidence that this additional membrane serves as a reservoir of membrane which is inserted in the CM during spore germination to allow core expansion.

## Results

### Cores of dormant *Bacillus subtilis* spores contain intracellular membrane-like structures just below the core membrane (CM)

**Figure 2:**
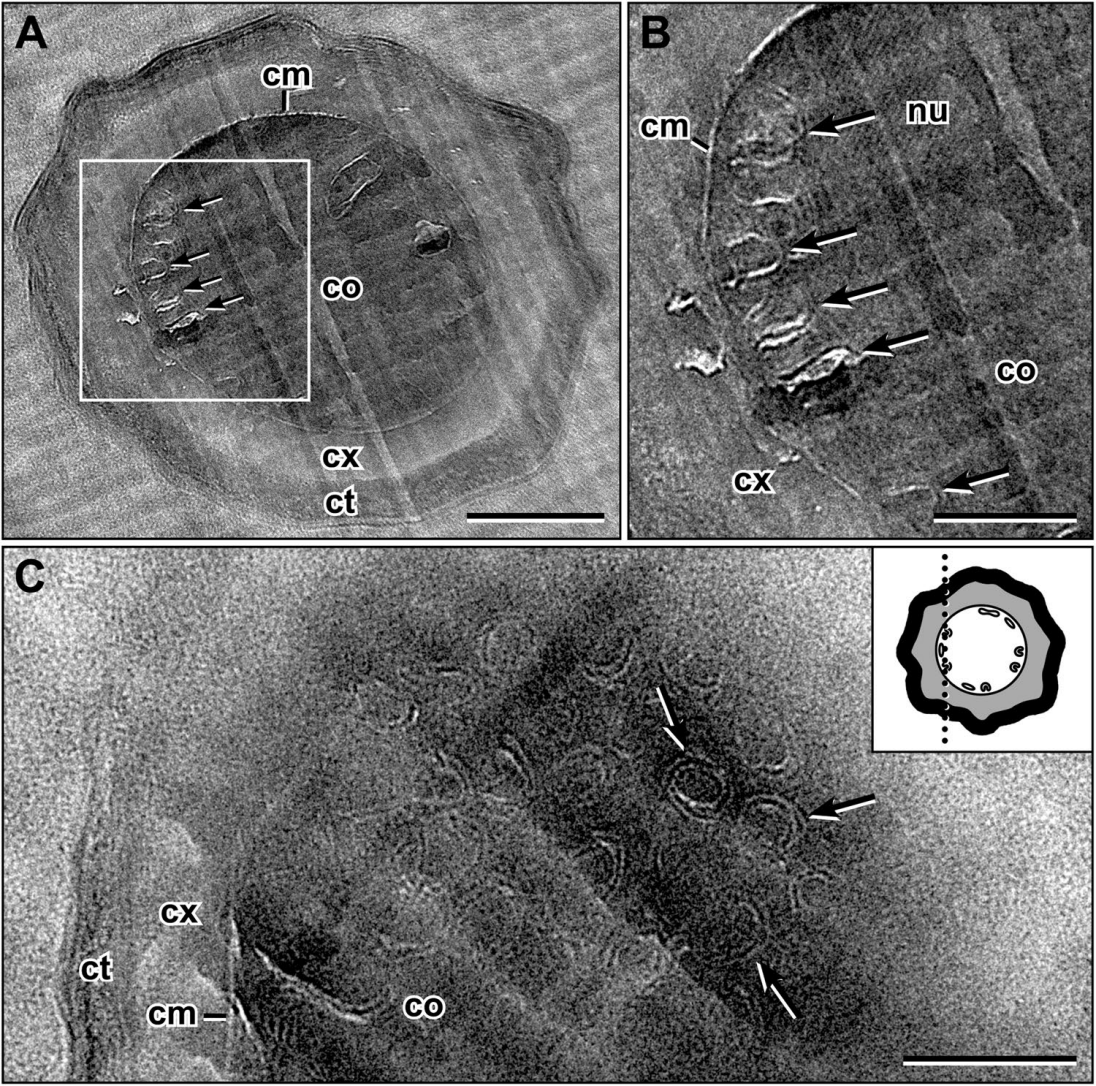
Cryo-electron microscopy of vitreous sections (CEMOVIS) through dormant spores of *B. subtilis*. (**A**) Cross-section through an entire spore, which shows sub-core membrane membranes (sCMMs; arrows) in the core, directly below the CM (cm). (**B**) Enlarged view of the sCMMs of the boxed core area in (**A**). sCMMs mostly show a periodic dark-bright-dark-bright-dark contrast. (**C**) Partial view of a tangential section through a dormant spore almost parallel to the long axis of the spore and directly below the inner surface of the CM (see inset for a schematic illustration of the level of sectioning). At this section plane, numerous double-ring sCMMs with the periodic dark-bright-dark-bright-dark contrast are visible. Abbreviations: cm = core membrane, co = core, ct = coat, cx = cortex, nu = crystalline nuleoid^9^, scale bar in (**A**) = 200 nm, and in (**B**) and (**C**) = 100 nm.

Cryo-electron microscopy of vitrified sections (CEMOVIS) through dormant spores of *B. subtilis* revealed membrane-like structures within the core directly below the CM (Fig. 2). These sub-CM membranes (sCMMs) appeared as loops, with an opening towards the CM, or as short lamellae (Fig. 2A,B; Supplementary Fig. S1). The sCMMs showed either a dark-bright-dark contrast, which is similar to that of the CM, or a periodic contrast (dark-bright-dark-bright-dark) (Fig. 2B,C; Supplementary Fig. S1). Tangential sections through spores parallel to the long axis resulted in section levels parallel to the CM surface and revealed double-ring structures with a periodic contrast (Fig. 2C). Thickness measurements of the sCMMs resulted in a mean width of 5.4 nm (n = 14) for the structures with a single dark-bright-dark contrast, which is identical to the mean width measured for the CM (5.4 nm, n = 21). sCMMs with a periodic contrast revealed a mean width of 9.9 nm (n = 28), which is about 1.8-fold greater than the mean CM width. Representative images from the membrane thickness analysis are shown in Supplementary Fig. S1. The sCMMs’ structural appearance, i.e. contrast, shape, and size suggests that the sCMMs with a single dark-bright-dark contrast are single membranes such as the CM and that the sCMMs with a periodic contrast could represent two layers of closely apposed membranes.

**Figure 3:**
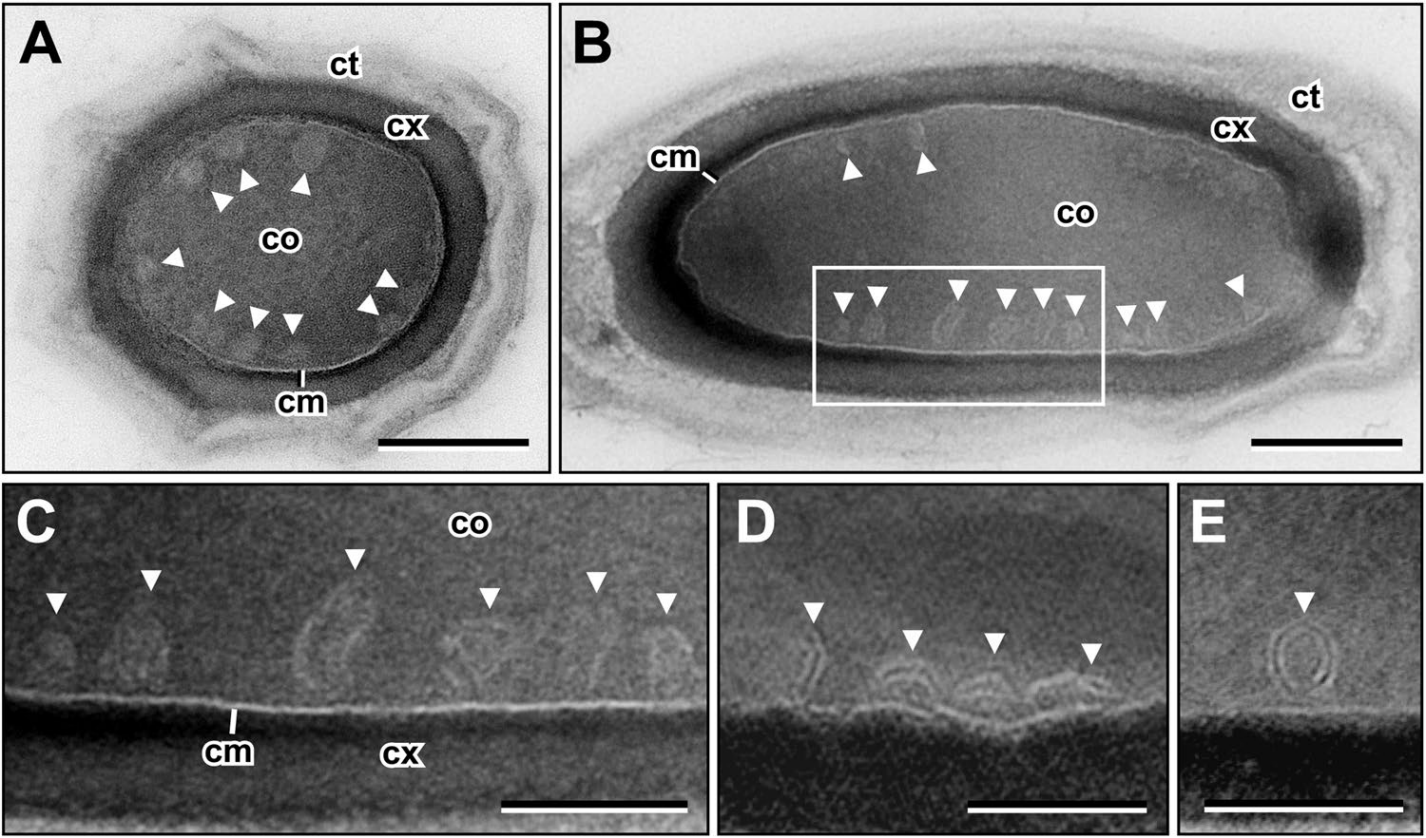
Conventional thin section electron microscopy of dormant *B. subtilis* spores. (**A**) Cross-section and (**B**) longitudinal section. sCMMs (arrowheads) appear as round or ellipsoid tubulo-vesicular spots in the core below the CM (cm) and only partially reveal their membrane-like boundary. (**C**) Enlarged view of boxed area in (**B**) which shows that the membrane-like morphology is only partially visible and that the sCMMs exhibit a dark-bright-dark contrast. (**D**,**E**) Tomography slices show that sCMMs (arrowheads) appear also in form of lamellae and as double-rings with the periodic contrast (dark-bright-dark-bright-dark) which was detected by CEMOVIS. Abberviations: cm = core membrane, co = core, ct = coat, cx = cortex, scale bar in (**A**), (**B**) = 200 nm, and in (**C**–**E**) = 100 nm.

To facilitate further structural and functional analysis of the sCMMs within the core, we tested if we could find them in chemically fixed, plastic embedded spores. Cryo-preparation methods, such as high-pressure freezing and freeze-substitution, do not preserve the core of dormant spores^9^. Microwave-assisted chemical fixation of dormant spores, embedding in LR White and the application of a particular section staining procedure, employing uranyl acetate/methyl cellulose, allowed the preservation and visualization of sCMMs within the spore core (Fig. 3) while embedding in Epon or LR White followed by conventional uranyl acetate and lead citrate contrasting did not. However, the sCMMs in sections from LR White preparations did not reveal the distinct contrast pattern which could be seen for the sCMMs using CEMOVIS. Although the CM appeared as a distinct white line surrounded by darker lines (similar to the contrast shown by CEMOVIS), the internal sCMMs appeared as thin white lines often without a sharp contrast (Fig. 3B,C). In thicker sections (i.e. 80–90 nm) sCMMs appeared as circular regions of lower density than the rest of the core directly below the CM which might represent circular sectioning profiles of round or tubular 3D structures (Fig. 3A, Supplementary Fig. S2). Since the visibility of the sCMMs seemed to correlate with the section thickness (the thinner the section, the sharper the contrast), we recorded tomographic tilt series of thin sections and computed tomograms. In digital sections of such tomograms, the sCMMs mostly showed the periodic contrast which was already observed by CEMOVIS and which suggests the presence of closely apposed double-membrane layers (Fig. 3D,E; Supplementary Video 1). Unfortunately, we were not able to achieve suitable tomograms from thicker (>90 nm) sections, even by using higher acceleration voltage (i.e. 200 kV), and this precluded a 3D reconstruction of the sCMMs in a volume large enough to contain a significant portion of the entire 3D structure. However, we never observed a direct membrane connection between the spore core membrane and sCMMs, something which would be expected in case of a persisting invagination of the core membrane.

**Figure 4:**
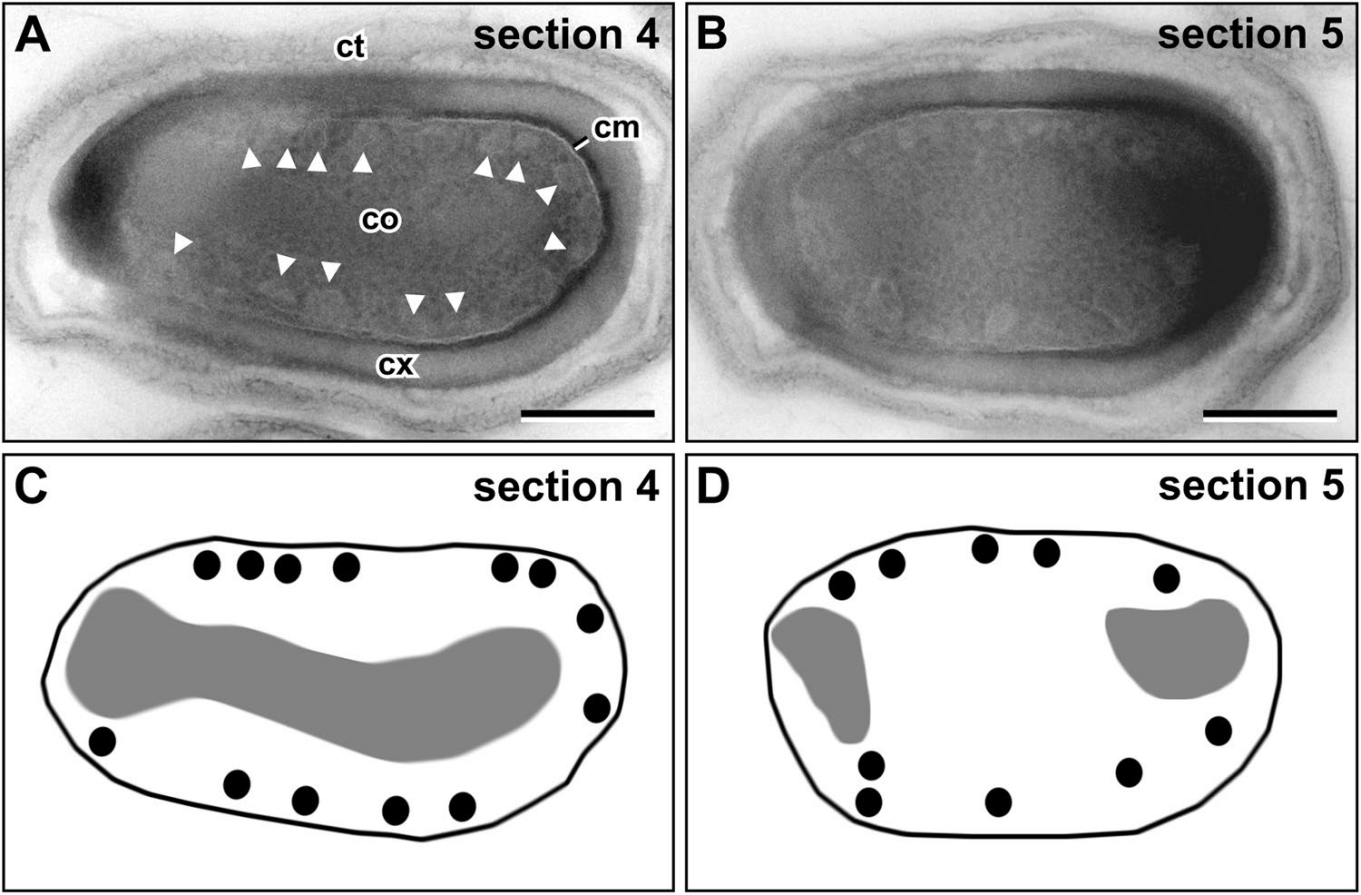
Distribution of sCMMs (arrowheads) within the core (co) of dormant *B. subtilis* spores in serial sections. (**A**) Section 4 and (**B**) section 5 of 10 serial sections through a dormant spore (see Supplementary Fig. S3 for all sections through the spore). (**C**,**D**) Schematic representation of the localization of sCMMs (black dots) within the spore. sCMMs show no particular concentration in a specific core region but are not densely packed. In regions where the nucleoid (grey areas) approaches the CM (cm), sCMMs are absent. Abberviations: cm = core membrane, co = core, ct = coat, cx = cortex, scale bars = 200 nm.

The sCMMs could be found in almost all (97%; n = 100) thin (70–80 nm) sections of LR White embedded dormant spores, which reveal a core structure (mechanically disrupted or covered spore sections were excluded). To obtain information about the spatial localization of the sCMMs within the spore core, serial sectioning of spores (n = 10) was performed. The analysis of serial sections taken parallel to the spore long axis, revealed no preferred localization of the sCMMs below the CM. Indeed, the sCMMs were rather homogenously distributed, although did not follow the entire CM surface (Fig. 4; Supplementary Fig. S3). In particular, in some regions the core nucleoid approached the CM, obviously leaving no space for the sCMMs (Fig. 4). In other regions devoid of sCMMs, no other special structures could be observed (Fig. 4). However, tangential sections through the core reveal rather uniformly and densely distributed circular section profiles of sCMMs (Supplementary Fig. S2), which supports the conclusion of a relatively ubiquitous presence of sCMMs below the CM of dormant spores.

### sCMMs are labelled by an antibody against the abundant CM protein SpoVAD

**Figure 5:**
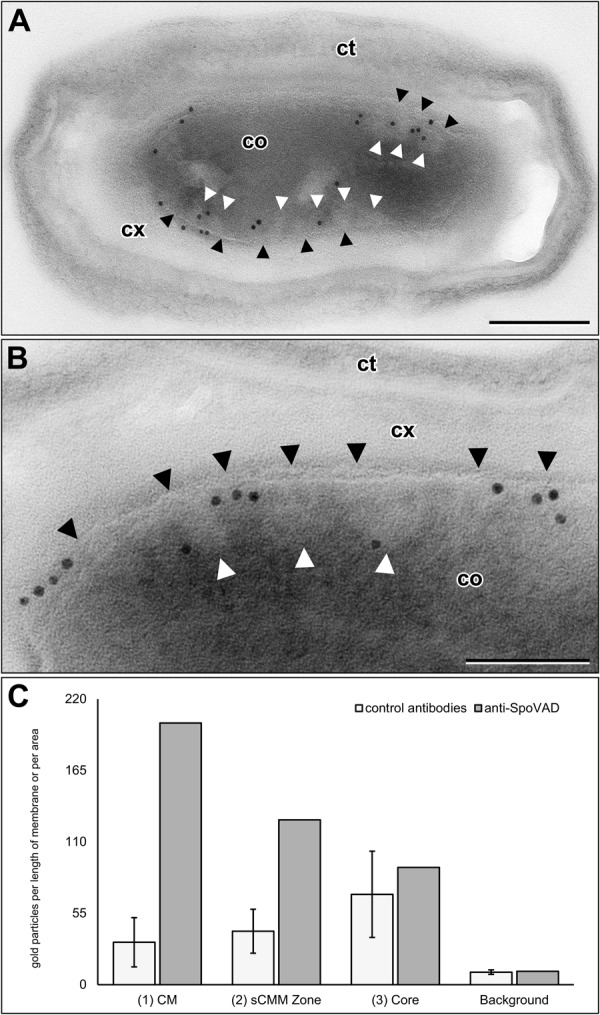
Immunogold labelling of sections through dormant spores of *B. subtilis* with an affinity-purified antibody raised against SpoVAD. (**A**) Longitudinal section through a spore which shows the general distribution of the gold particle labelling. Significant labelling is seen along the CM (black arrowheads) and in the zone below the CMc where the sCMMs (white arrowheads) are localized. Note that the visibility of sCMMs is reduced in comparison to Figs 3 and 4 but their identification still is possible due to the reduced electron density of the structures in comparison to the central part of the spore core. (**B**) Detailed view of the labelling of the CM zone and the adjacent zone with three sCMMs (white arrowheads). The CM (black arrowheads) is strongly labelled and two of the three sCMMs (white arrowheads) are labelled. (**C**) Quantification of the labelling densities of the anti-SpoVAD labelling (grey bars) in comparison to labelling with three control antibodies (white bars) for the background and different core regions: (1) CM, (2) sCMM zone, (3) core, (see Supplementary Fig. S6). Labelling densities of the three control antibodies are expressed as the mean of the labelling density distribution and error bars correspond to the SD. Anti-SpoVAD labelling density of the CM and sCMM zone is at least 5-fold SD higher than the mean labelling density of the control antibodies which implies specific labelling. In contrast, core labelling is considered nonspecific because difference between labelling densities of anti-SpoVAD and control antibodies is below 1 SD (see also Table 1). Abbreviations: cm = core membrane, co = core, ct = coat, cx = cortex, scale bar in (**A**) = 200 nm and in (**B**) = 100 nm.

To test whether the sCMMs are membranes like the CM, we used an antibody raised against an abundant CM protein, SpoVAD, in immunogold labelling experiments on plastic sections through dormant spores. These labelling experiments showed that the CM and the zone below the CM, which contains the sCMMs (see Fig. 3), were strongly labelled while the core region below the sCMMs, as well as the cortex and coat showed little or almost no labelling (Fig. 5). Although the visibility of sCMMs decreased significantly during immunolabelling procedures, association of gold labelling with structures similar in size, shape and localization to sCMMs of the core was possible (Fig. 5B). Quantitation of the anti-SpoVAD labelling in comparison to the labelling of three control antibodies clearly suggested that the anti-SpoVAD labelling of the CM and of the adjacent core zone, in which the sCMMs are localized, was specific, because labelling with anti-SpoVAD in those regions was significantly higher (difference at least 5-fold standard deviation of controls) than with the control antibodies (Fig. 5C; Table 1). In contrast, the observed labelling of the core was almost identical with anti-SpoVAD and control antibodies (difference was smaller than one standard deviation of controls) (Fig. 5C; Table 1) and thus was considered as nonspecific (see Materials and Methods for a detailed description of the specificity settings).

**Table 1:** Quantification of anti-SpoVAD immunogold labelling of sections through dormant *B. subtilis* spores.

Antibodies	Labelling density on spore structures			
	(1) CM [part/50 µm]	(2) sCMM [part/µm^2^]	(3) Core [part/µm^2^]	Background [part/µm^2^]
arPBP (control 1)	17	28	37	8
aAcz2 (control 2)	59	65	115	9
aGFP (control 3)	22	30	57	12
**control (mean)**	**33**	**41**	**70**	**10**
SD (control)	19	17	33	2
**aSpoVAD**	**202**	**127**	**90**	**10**
**Difference between aSpoVAD and control (aSpoVAD-control)/SD**	**8.9**	**5.1**	**0.6**	**0.4**

Labelling densities (gold particles [part] per membrane length or core area) of three different core zones (CM, sCMM, core) and the background labelling of the coats were determined for anti-SpoVAD and three control antibodies. For each spore region, the difference between anti-SpoVAD labelling density and mean labelling density of control antibodies in this same region was calculated and expressed relative to the standard deviation (SD) of the control labelling. The difference of labelling density is above 5 SD in zone (1) and (2) and close to 1 SD for core zone (3) and the background indicating specific labelling of the zones containing the CM and the sCMMs, and nonspecific labelling of the core zone (3) which is devoid of membranes.

### sCMMs disappear during early stages of germination

**Figure 6:**
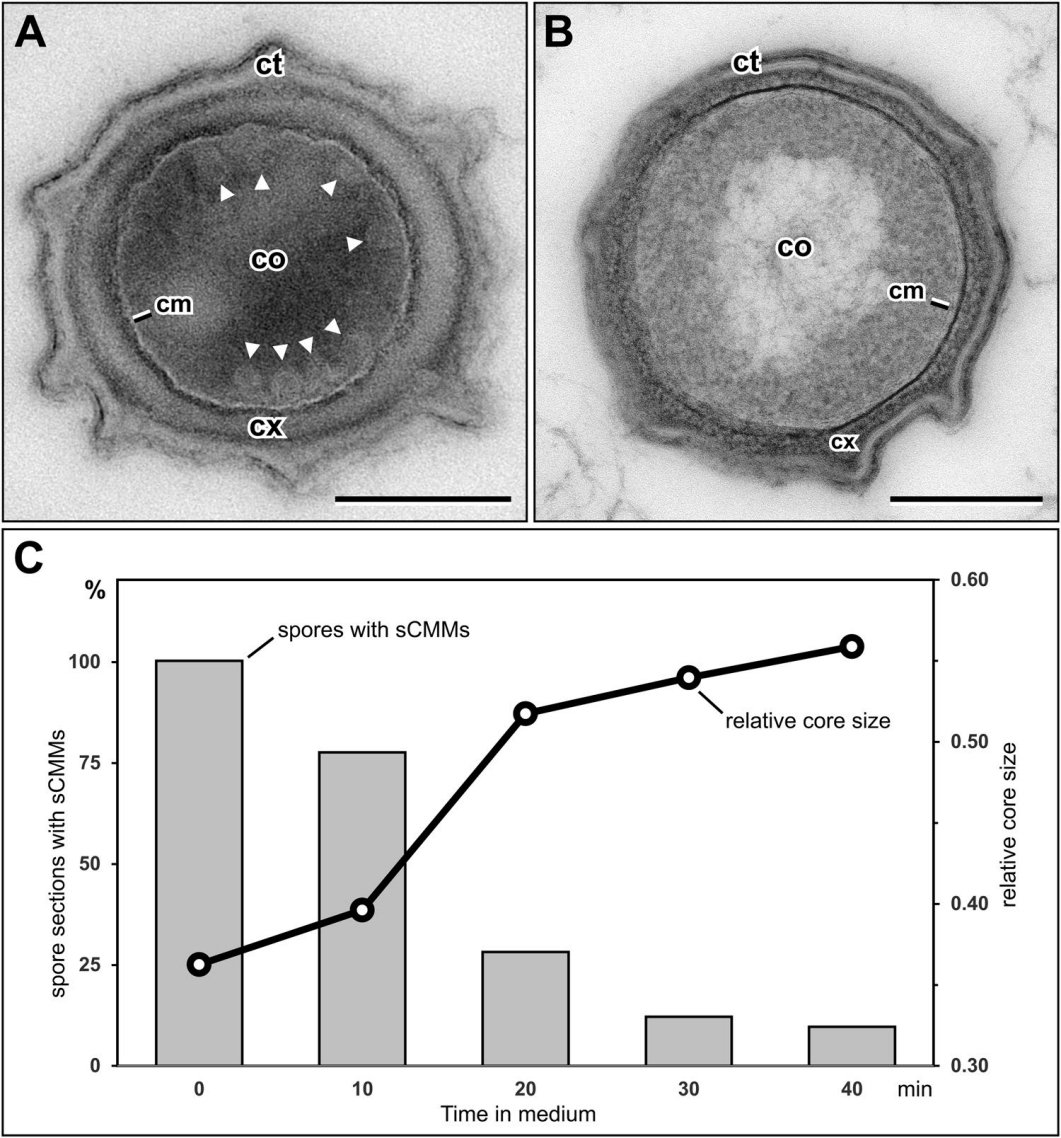
Presence of sCMMs in spores of *B. subtilis* during germination. Cross-section through a dormant spore (**A**) and a germinating spore (**B**) which was incubated in TSB for 20 min. While sCMMs (arrowheads) can be seen in the dormant spore, the section through the germinating spore does not reveal any membrane structures with a similar morphology and location in its core. As expected, the core (co) of the germinating spore is much larger than the core of the dormant spore and the cortex (cx) appears thinner, less compact and rather filamentous. (**C**) Relative core size and presence of sCMMs in sections through spores fixed at different times of spore germination in TSB medium (30 sections per time point were evaluated). The diagram shows an inverse correlation between relative core size (core circumference divided by spore circumference) and presence of sCMMs over time of germination. The fraction of spore sections showing sCMMs drops over time as the relative core size increases. Most remarkably, the steepest drop in the percentage of spores with sCMMs is associated with the steepest increase in relative core size (between 10 and 20 min). See also Supplementary Fig. S4 for the time course of this germination experiment in phase-contrast light microscopy which shows that practically all spores had completed the transition from phase-bright to phase-dark during this period indicating at least the beginning of cortex degradation and core expansion (stage II of germination). Abbreviations: cm = core membrane, co = core, ct = coat, cx = cortex, scale bars = 200 nm.

Morphological features of the sCMMs, such as contrast and width, and the localization of an abundant CM protein in them, indicate that these structures are membranes, like the CM. To get an idea about the function of the sCMMs, we analyzed their fate in germinating *B. subtilis* spores. Spore germination in rich TSB medium was stopped at different time points by adding concentrated fixative. After embedding in LR White resin and ultrathin sectioning, samples were investigated by transmission electron microscopy. As expected, dormant spores showed sCMMs (Fig. 6A). With ongoing germination, the number of spores which show structural signs of germination, such as disintegration of the cortex and core swelling, increased. Moreover, the sCMMs disappeared (Fig. 6B). To get more precise information about the time course of sCMM disappearance, we measured the presence of sCMMs in relation to the core size during germination. While the relative core size (core circumference relative to outer spore circumference) in spore sections increased with germination time, the fraction of spore sections showing sCMMs decreased. Remarkably, the steepest drop in the percentage of spore sections showing sCMMs occurred between 10 and 20 min of germination, exactly when the steepest increase in relative core size could be measured (Fig. 6C). The observed increase of the relative core size in this period is in accordance with the results from phase-contrast microscopy, which showed that between 10 and 20 min most of the spores changed their appearance from phase-bright to phase-dark (Supplementary Fig. S4), indicating completion of stage I of germination marked by the release of most (~70%) of the CaDPA and at least the beginning of stage II in which cortex degradation and core expansion take place^5,11^. In summary, the obvious correlation between core expansion and disappearance of sCMMs suggests that the sCMMs are incorporated into the CM during core expansion in spore germination.

### sCMMs are also present in spores of other endospore forming bacteria

**Figure 7:**
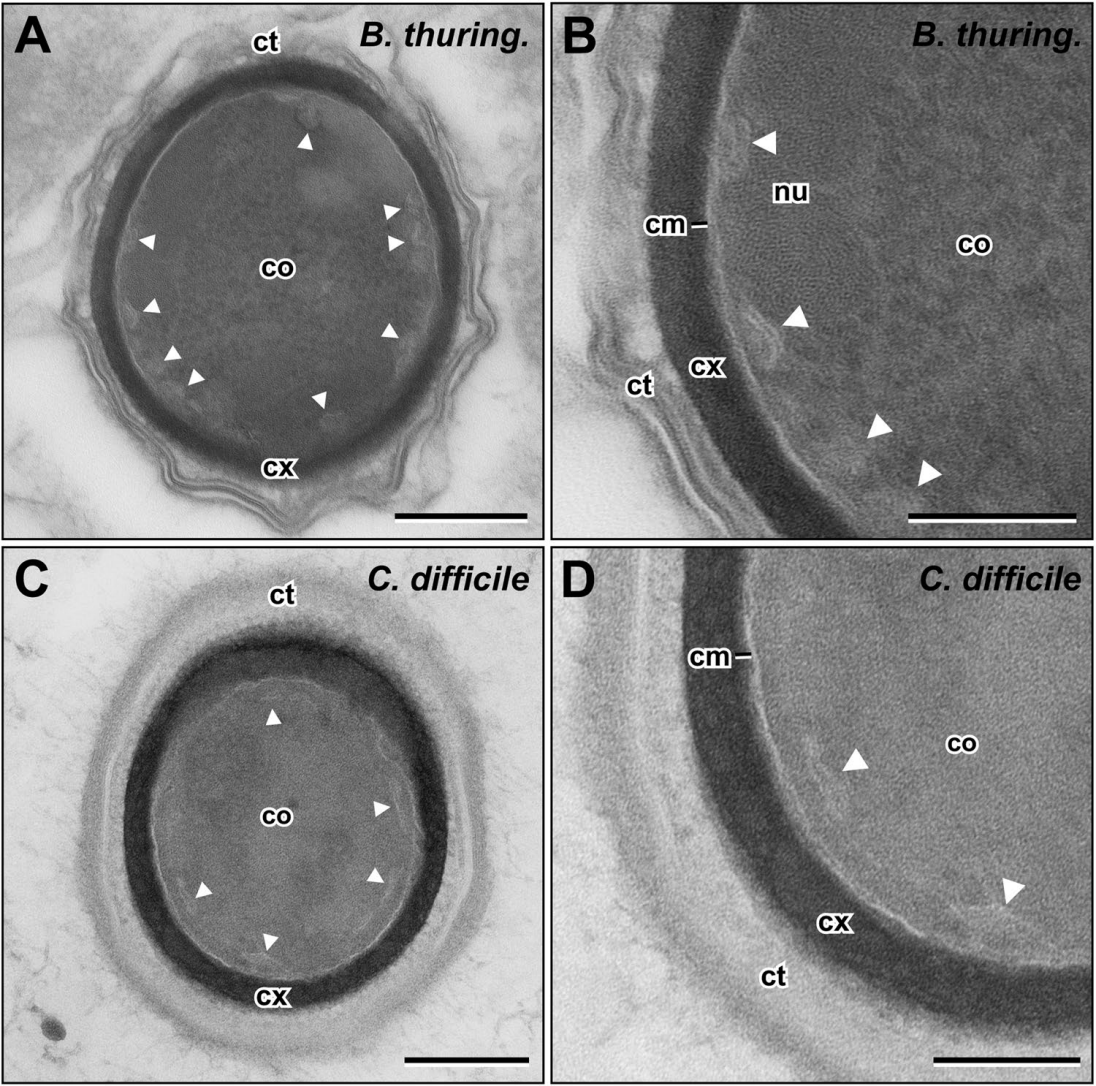
sCMMs are present in spores of different Firmicute species. (**A**) *B. thuringiensis* and (**C**) *C. difficile*. (**B**,**D**) show respective enlarged views of sCMMs (arrowheads). See Supplementary Fig. S5 for images of more species. Abbreviations: cm = core membrane, co = core, ct = coat, cx = cortex, nu = nucleoid, scale bars in (**A**), (**C**) = 200 nm, and in (**B**,**D**) = 100 nm.

To investigate whether the sCMMs in the core of dormant spores are a special feature of *B. subtilis* spores, we analyzed spores of other endospore forming bacterial species: *Bacillus thuringiensis, B. anthracis* Sterne*, Geobacillus stearothermophilus, Clostridioides difficile* and *Clostridium botulinum*. In spores of all of these species we could detect the sCMMs in the spore core within a distinct zone directly below the CM (Fig. 7, Supplementary Fig. S5). While the sCMMs in *B. anthracis* (Supplementary Fig. S5A) and *B. thuringiensis* (Fig. 7A,B) spores were similar to the sCMMs found in *B. subtilis* spores (Fig. 2A,B), intracellular membranes in the spores of Clostridia and *Geobacillus* appeared more tubular (Fig. 7C,D, Supplementary Fig. S5B, C). However, sCMMs could be found in the core of spores at similar locations in all species studied.

## Discussion

Thin section electron microscopy visualized sCMMs in the core of *B. subtilis* spores in a zone directly below the CM. The presence of these sCMMs could be demonstrated by cryo-electron microscopy of vitreous sections (CEMOVIS), which eliminates artifacts introduced by chemical processing^12^ and by conventional electron microscopy using chemical fixation and plastic embedding. In CEMOVIS the sCMMs had similar structural features (contrast, width) as the CM. Visualization of sCMMs in plastic-embedded samples was possible by using the hydrophilic acrylate resin LR White in combination with a particular staining approach for sections^13^ which provides a mixture of positive and negative contrast. Interestingly, internal core membranes similar to the sCMMs could be demonstrated in spores of *B. popilliae* without analyzing them further by using potassium permanganate and osmium tetroxide as fixatives^14^, which resulted also in a mixture of positive and negative contrast, at least within the core. Conventional thin section electron microscopy using epoxy resin embedding and contrasting of sections with uranyl acetate and lead citrate provides rather positive contrast^15^ and did not allow visualization of sCMMs. Since this procedure is generally used for the analysis of spore ultrastructure, it appears obvious that the sCMMs have been widely overlooked so far. However, even in the negatively stained LR White sections, visualization of sCMMs was impaired in comparison to visualization by CEMOVIS and did not reveal the morphological membrane features of all sCMM structures. The visualization of sCMMs was dependent on section thickness and staining quality (i.e. thickness of the uranyl acetate/methyl cellulose film) although the CM usually was clearly visible. This discrepancy in membrane visibility can be explained by the different environmental conditions for the CM and the sCMMs. In the dormant spore, core molecules and supramolecular structures (e.g. the nucleoid), including the sCMMs, are surrounded by rather solid and immobile CaDPA^16,17^ which most likely is preserved during our comparatively rapid preparation for thin section electron microscopy and this could interfere with the penetration and/or binding of the contrasting chemicals. In contrast, CaDPA is only present on one side of the CM, the core side, while the side facing towards the cortex is at least accessible for small molecules activating the germination receptors^5^.

Apart from the morphological features, two other results indicate that the sCMMs are bio-membranes like the CM. First, the abundant CM protein, SpoVAD, is also localized in the sCMMs and second, the sCMMs disappear in spore germination exactly when the spore core expands due to water influx and cortex hydrolysis. SpoVAD is involved in the uptake of CaDPA from the mother cell where CaDPA is synthesized, and for release of CaDPA from the core during spore germination^5^. This protein is synthesized in the developing spore during sporulation and there are ~15,000 molecules of this protein per spore, and largely in the membrane fraction isolated from disrupted spores^18^. Presumably this membrane fraction includes both CM and sCMMs. Immunogold labelling with an affinity-purified antiserum raised against SpoVAD from *B. subtilis*^18^ resulted in a dense and specific decoration of the CM zone (width 20 nm) and the adjacent core region (width 40 nm) which contains the sCMMs. The labelling in both zones by the anti-SpoVAD antibody was considered as specific based on a comparison with labelling by three control antibodies and therefore most likely represent the localization of SpoVAD. Displacement of SpoVAD molecules from the CM into the sCMM zone caused by extraction during sample preparation, or displacement of the antibodies decorating the membrane, seem unlikely reasons for the observed labelling pattern because the cortex was not significantly labelled (Fig. 5A,B). Extraction and label displacement should be more uniform and not only directed towards the core. However, we cannot fully exclude the influence of these factors on the labelling result. It is also remarkable that the central core zone itself showed some labelling which was considered as rather nonspecific because control antibodies produced almost the same labelling density (Fig. 5C). However, it is possible that some SpoVAD molecules, which are quite hydrophilic^19^, are not incorporated into the SpoVA protein complex in spore membranes during sporulation and still reside within the core plasma. In any event, the labelling results indicate that SpoVAD is localized not only in the CM, but also in the sCMMs supporting the sCMMs’ identity as bio-membranes.

**Figure 8:**
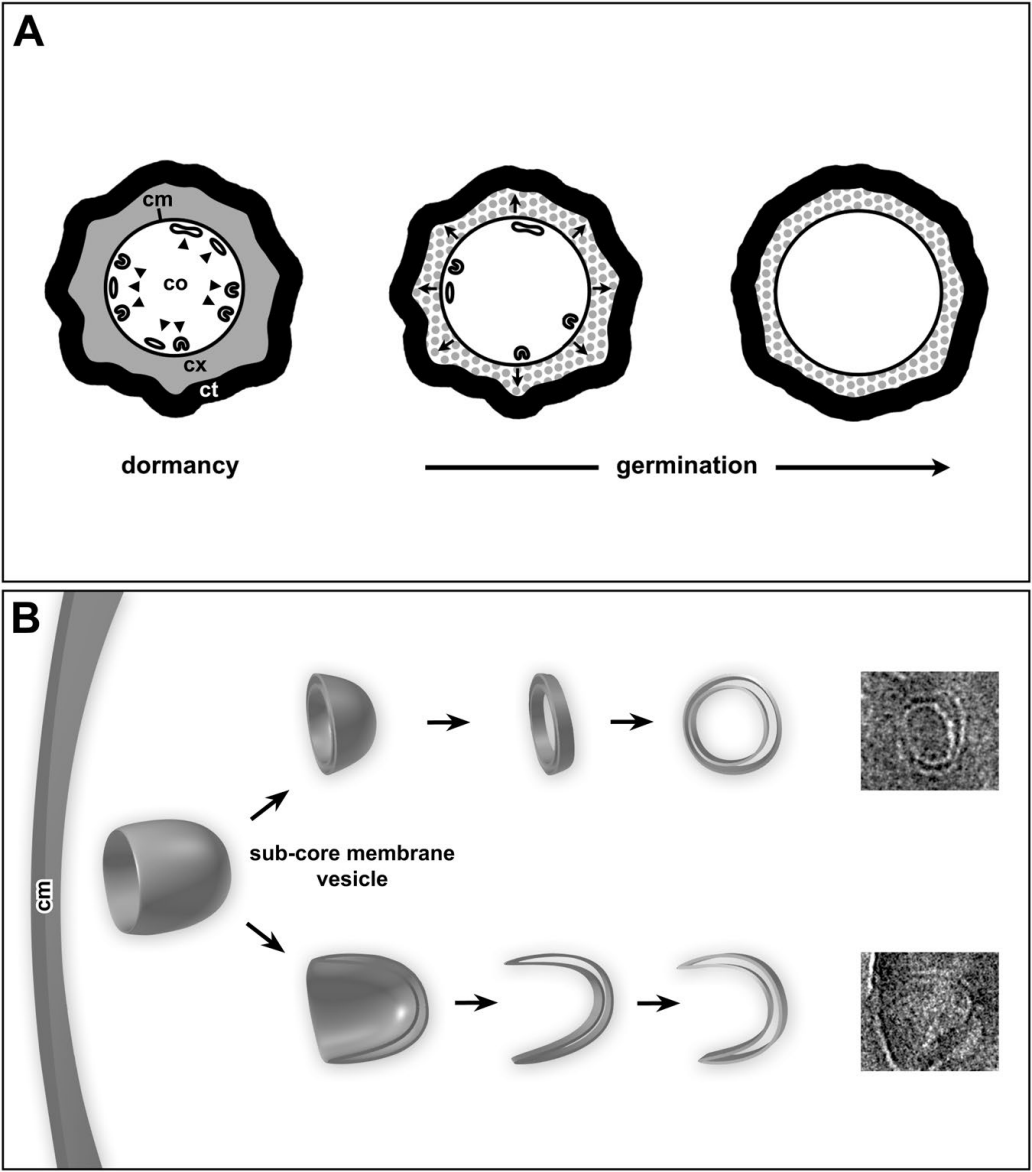
(**A**) Schematic representation of the morphological events in spore germination. The sCMMs (arrowheads) disappear in a period when core (co) expansion (arrows) and cortex (cx) lysis is taking place, consistent with sCMM integration into the CM (cm) during germination to allow core expansion. (**B**) Schematic diagram of the presumed 3D morphology of sCMM structures in dormant *B. subtilis* spores as deduced from CEMOVIS data. A vesicular shape with a cup-like compressed membrane surface at one side would be sufficient to explain at least part of the sectioning profiles found and which are shown on the right side.

Core expansion is a morphological hallmark of stage II during spore germination, in which full spore core rehydration and metabolic activity is restored^6,11^. It has been unclear how the CM is able to allow the ~1.3- to 1.6-fold expansion in CM surface area required for core volume expansion without any lipid biosynthesis^7^. Our morphological analysis of germination clearly shows that the relative core size in thin sections is increasing while at the same time the fraction of spore sections revealing sCMMs is decreasing. This correlation is particularly obvious between 10 and 20 min after initiating germination in our experiments when almost all spores turned from phase-bright to phase-dark in phase-contrast microscopy. Since we did not conduct continuous measurements of the refractility of individual spores during germination, we can only conclude that the major part (~ 70%) of the refractive index change from phase-bright to phase-dark was accomplished^20^. However, this indicates that CaDPA was fully released by the spores and that the spore most likely entered stage II of germination and beginning cortex hydrolysis and core expansion^6,11^, which supports our observation of an increase in core size during this period of germination. The most intuitive explanation for the disappearance of the sCMMs is that these membranes were incorporated into the CM to facilitate the increase in CM surface area accompanying core expansion (Fig. 8A). From the serial section analysis (Fig. 4) we can conclude that at least 30% of the inner core membrane surface is accompanied by sCMMs. If we simply assume that the sCMMs are present as a compressed vesicle (see below), this would at least result in a doubled membrane surface for the sCMMs along the CM which would correspond to about 60% of the entire CM surface and be sufficient to explain the 30 to 60% of CM area increase during germination. However, as yet we have been unable to visualize the integration of the sCMMs into the CM. Membrane fusion processes are extremely rapid^21,22^ and difficult to capture. Perhaps experiments at a higher temporal resolution regarding the time course of germination and increasing the speed of fixation (e.g. by using cryofixation) could help to increase the likelihood for finding membrane fusion events by thin section electron microscopy. Since the sCMMs are localized in a zone below the CM, a molecular link between the CM and sCMMs seems likely. However, it is unclear how these two types of membranes are linked.

Our analysis of the presence of sCMM in spores of different Bacillales and Clostridiales species suggests that sCMMs are a universal feature of spores. This is not unexpected, since much of the spore germination machinery and architecture providing spore resistance are universal in all Firmicute spore-formers^2,5^. Indeed, pan-species universality was recently shown for the crystalline organization of the DNA in the dormant spore which is an important factor for providing DNA resistance^9^. However, while sCMMs were found in spores of all species, their section profiles were somewhat different in different species. In spore of *B. subtilis*, *B. anthracis* and *B. thuringiensis*, sCMMs were more vesicular, but in spores of *G. stearothermophilus*, *C. difficile* and *C. botulinum* they appeared more tubular.

Ultimately, analysis of the sCMM 3D structure at higher resolution is needed to get more information about these membranes and their potential link to the CM. Cryo-tomography of slices prepared by cryo-focussed ion beam milling^23–25^ may help to solve this issue when this technique becomes more widely available. So far, we can only speculate about the 3D structure of the sCMMs. The most intuitive fit with the sectioning profiles detected by CEMOVIS (this technique gives the most reliable structural representation) suggests a 3D structure of a compressed vesicle or tubule (Fig. 8B). This hypothesis would be in accordance with known membrane dynamics in cells mediated by fission and fusion of vesicles or small tubules. A potential compression of sCMM bound structures can be explained by the exceptional dryness of the core in association with the deposition of CaDPA which together may deform small membrane-bound compartments like vesicles or tubules. Future investigation should also focus on the formation of sCMMs during sporulation to understand their positioning and shape within the dormant spore.

## Materials and Methods

### Bacterial strains and spore production

*B. subtilis* subsp. *spizizenii* (ATTC 6633) was cultivated in Luria-Bertani (LB) medium for about 18 h at 37 °C on a rotary shaker at 200 rpm. For sporulation, this culture was diluted 1:10 in sporulation medium^26^ (the glutamic acid concentration was reduced 10-fold) and cultivated for another 8–15 days at 37 °C and shaking at 200 rpm. Sporulation was monitored by inspection with phase-contrast microscopy. After reaching sporulation in more than 95% of the bacterial cells, the culture was centrifuged (2000 g, 10 min, 4 °C). The resulting pellet showed three layers from which the uppermost layer was harvested and suspended with 0.05 M Hepes buffer (pH 7.4). After two further centrifugation and resuspension steps with Hepes buffer, the suspension was stored at 4–8 °C. The purity of the suspension was checked by phase-contrast microscopy and levels of spore particles were determined using a hemocytometer according to Neubauer. *B. thuringiensis* (DSM 350) spores were produced following the same protocol. Suspensions of spores prepared according to this procedure contained only few (<=1%) residual vegetative bacteria.

*Geobacillus stearothermophilus* (NCIB 8923 = ATCC 12980) was cultivated on solid LB agar for three days at 56 °C. For sporulation, the agar plates were transferred to room temperature. The progress of sporulation was monitored by phase-contrast microscopy and took a few days to reach significant values. Cells were harvested using a Drigalski spatula and were filtered through glass wool before they were washed three times with deionized water to remove vegetative cells or debris. Purified spores were stored in 70% ethanol at 4–8 °C.

Spores of *B. anthracis* Sterne (strain 34F2), *Clostridioides difficile* (NCTC 13366) and *Clostridium botulinum* (NCTC7272) were produced according to European standard procedures (prEN 14347:2001 [D]) and stored at 4–8 °C in deionized water or in 70% ethanol until use.

### Cryo electron microscopy of vitreous sections (CEMOVIS)

Spore pellets of *B. subtilis* were frozen by self-pressurized rapid freezing, sectioned at low temperature and imaged by cryo-transmission electron microscopy as described previously^9^. The widths of the CM and the sCMMs were determined by using the “Plot Profile” function of ImageJ^27^. Before analysis, images were adjusted to similar contrast/brightness and filtered with a mean filter (radius = 1.5 pixel). With the line tool a short line (approximately 20 nm) was drawn across the membrane at an approximately 90° angle to the membrane surface and the “Plot Profile” function was executed. The resulting line plot (grey level relative to the position on the selection line) was used for measuring the distance between minima (darkest parts) of the selected profile which correspond to the outer (electron dense) parts of the CM (Supplementary Fig. S1). Line profiles across double-ring membrane-like structures revealed three minima, and the distance between the outer two minima was determined (Supplementary Fig. S1).

### Conventional thin section electron microscopy of plastic-embedded spores

The procedures for conventional thin section electron microscopy were described in detail previously^9^. Briefly, spore pellets or suspensions were chemically fixed in 10% formaldehyde, 0.05% glutaraldehyde in 0.05 M Hepes buffer (pH 7.4) using a microwave-assisted protocol which heated the spore/fixative mixture up to 50 °C within 5 min. After embedding in an agarose gel, samples were quickly dehydrated and embedded in LR White which then was polymerized on ice by the help of an accelerator. Ultrathin sections (60–90 nm) were produced with an ultramicrotome (Leica EM UC7; Leica Microsystems, Germany) and collected on slot grids. Sections were stained with a solution of 0.9–1.8% uranyl acetate and 0.1% methyl cellulose (w/v) for 10–20 min^13^ and examined using a transmission electron microscope (Tecnai 12 Spirit; FEI, USA) at 120 kV. Images were recorded with a CCD camera, either at a resolution of 1376 by 1024 pixels (Megaview III; Olympus SIS, Germany) or 4096 by 4096 pixels (Eagle; FEI, USA).

### Electron tomography

Single axis tilt series (−70 to +70° at 1° increments) were recorded from ultrathin plastic sections (70–150 nm) using a Tecnai 12 Spirit (FEI, USA) transmission electron microscope operated at 120 kV. Image acquisition was controlled by the FEI tomography software and performed with a 2 K CCD camera (F-216, TVIPS, Germany). To facilitate image alignment, sections were coated with gold particles at the back side of the supporting film. Tomograms were computed with the Inspect3D software (FEI, USA) using the SIRT algorithm (25 iterations).

### Immunogold electron microscopy

Slot grids with ultrathin sections (70–80 nm thick) of the LR White embedded *B. subtilis* spores were incubated on droplets (30 µl) at room temperature using the following protocol: 2x glycine (50 mM) in PBS for 5 min; blocking solution (0.5% fish gelatin, 0.5% bovine serum albumin [protease free; T844.1; Carl Roth GmbH, Germany], and 0.01% Tween 20 [Fisher Scientific] in PBS) for 1 min; blocking solution for 15 min; primary antibody diluted in blocking solution for 100 min; 6x blocking solution for 1 min each; secondary antibody in blocking solution for 30 min; two times blocking solution for 1 min each; glycine (50 mM) in PBS for 1 min; PBS for 1 min; four times deionized water for 1 min each. As primary antibodies, we used an affinity-purified antiserum raised against SpoVAD^18^ (anti-SpoVAD) and three control antibodies: (1) an antiserum raised against a recombinant insect pheromone-binding protein^28^ (anti-rPBP); (2) an affinity-purified antiserum raised against GFP (anti-GFP; Code 600-401-215; Rockland, USA); (3) an antiserum raised against an N-terminal part of the synaptic protein Aczonin/Piccolo^29^ (anti-Acz2). As secondary antibody, a goat anti-rabbit IgG (H&L) that was coupled to 10 nm gold (British Biocell International, United Kingdom) was used. After immunolabelling, sections were dried before they were contrasted using a solution of 0.9–1.8% uranyl acetate and 0.1% methyl cellulose (w/v) for 10–20 min^13^. Conventional transmission electron microscopy was done as described above.

Quantification of the immunogold labelling was done to assess specificity of the labelling. In this analysis, labelling by the anti-SpoVAD antiserum was compared with the labelling by the three control antibodies (anti-rPBP, anti-GFP, anti-Acz2), which are not known to detect any bacterial protein. For direct comparison, antibody concentrations of all antibodies were adjusted to give similar background levels over the coat layers, which most likely do not contain the target antigen SpoVAD^18^. Labelling densities (gold particles per membrane length or core area) were determined for three different core zones (Supplementary Fig. S6): (1) CM (+/−10 nm above and below); (2) sCMM zone (40 nm wide); (3) Core zone. The detailed procedures of sampling and determination of labelling densities are described in the Supplementary Methods section. The concept of this analysis is to determine the unspecific labelling of structures by control antibodies, which are not know to have any specific affinity for proteins in spores, and to use this labelling as a reference for the labelling with the specific anti-SpoVAD antibody. Any specific labelling must be different from the labelling achieved by the three control antibodies. The larger the difference is, the more likely the specificity of the labelling is. The variability of the labelling generated by the three different control antibodies, expressed as standard deviation (SD) of the mean labelling densities determined for each control antibody, provides a measure to assess the difference between control antibody labelling and anti-SpoVAD labelling. Usually a difference between two distributions is considered significant if the mean values differ at least by 2-fold SD which means that the two distributions would still overlap. A difference of about 5-fold SD exclude a significant overlap of distributions and is therefore considered as a robust criterion to conclude that there is a significant difference between two observed experimental conditions. In particle physics, the 5-fold SD threshold is widely used as a standard because of many false decisions using lower thresholds and it is discussed to use this strict threshold also in biomedicine^30^. We therefore computed the difference between labelling densities of anti-SpoVAD and control antibodies in units of the observed SD for the control antibody labelling and assessed the results according to the considerations stated above.

### Germination experiments

Spores of *B. subtilis* were transferred into deionized water and split into batches of 1 ml with 10^8^ spores in plastic reaction vials. Suspensions were centrifuged at 2000 × g for 10 min and the pellet was dried overnight at 37 °C. To initiate germination, dried spores were mixed with 1 ml of tryptic soy broth (TSB) using a vortex followed by incubation on a shaker (700 rpm; Thermomixer compact, Eppendorf) at 37 °C. At defined time points (see result section) spore suspensions (0.2 ml) were chemically fixed by mixing them with concentrated fixative (1 ml; final concentration: 10% formaldehyde, 0.05% glutaraldehyde in 0.05 M Hepes buffer). After 5 min of incubation at room temperature, fixed spore suspensions were heated in a microwave oven and processed for embedding in LR White as described above (Conventional thin section electron microscopy).

For phase-contrast light microscopy, 5 µl of the fixed suspensions were air-dried on a microscopic slide, covered with 10% polyvinylpyrrolidone and a coverslip. Microscopic investigation was done with an Axiophot light microscope (Carl Zeiss Microscopy, Germany) and the Plan-Neofluar 63×/1.25 oil immersion objective. Images were taken with a color CCD camera (Colorview II, OSIS, Germany) at 2576 × 1932 pixels (24 bit). Phase-dark and phase-bright spores (n > 200) were counted by using the multi-point tool of ImageJ^27^.

To determine the relative core size of dormant and germinating spores, section profiles of spores were randomly selected and photographed (20 cross sections and 10 longitudinal sections for each sample). Area measurements were done using iTEM software (OSIS, Germany). The outline of the entire spore section profile and of the core was labelled with the “Adaptive Polygon” tool and the respective areas were measured. Relative core size was calculated by dividing core area by the area covered by the entire spore section.

## Electronic supplementary material

Supplementary Video 1

Supplementary Information
