# Supplementary material for: Intracellular membranes of bacterial endospores are reservoirs for spore core membrane expansion during spore germination
Source: Sci Rep. 2018 Jul 30;8:11388. doi: 10.1038/s41598-018-29879-5 (PMC6065386; doi:10.1038/s41598-018-29879-5)
Supplement: Supplementary file 2 — Supplementary Information [file 41598_2018_29879_MOESM2_ESM.pdf]

Supplementary Information

Intracellular membranes of bacterial endospores are reservoirs for spore core membrane expansion during spore germination

Michael Laue[1*], Hong-Mei Han[2], Christin Dittmann[1], Peter Setlow[3]

[1] Advanced Light and Electron Microscopy (ZBS 4), Robert Koch Institute, Seestrasse 10, D-13353 Berlin, Germany

[2] Department of Systemic Cell Biology, Max-Planck-Institute of Molecular Physiology, Otto-Hahn-Strasse 11, D-44227 Dortmund, Germany

[3] Molecular Biology and Biophysics, UConn Health, Farmington, CT 06030-3305, USA

***Corresponding Author:**
Dr. Michael Laue
Advanced Light and Electron Microscopy (ZBS 4)
Robert Koch Institute
Seestrasse 10
D-13353 Berlin
Phone: ++49 (0) 30 18754 2675
e-mail: lauem@rki.de

Supplementary Methods

Quantification of immunogold labelling – Sampling and determination of labelling densities

Background labelling over spore coats was determined for randomized picked spore cross-sections (n = 20 sections for each antibody and dilution). Spore cross-sections with mechanical deformations (caused by the sectioning process) or impaired visibility of the coats (e.g. by particulate contaminations from the on-section contrasting) were excluded from the analysis. The area of the spore coats was determined by point and intersection counting (Griffiths 1993, page 415) using a grid width of 50 nm and the iTEM software (OSIS, Germany). Gold particles were counted manually. All data were collected in a spreadsheet, numbers for area and gold particles were summed for each data set and finally the particle density per area was computed and related to a reference area of 50 μm^2 to allow comparison with the particle densities determined for the core areas (see below).

For the determination of labelling densities on the core structures, spore section profiles (15 cross and 15 longitudinal sections) were randomly selected and photographed if they fulfilled the following criteria: i) CM visible (at least in two third of the section profile), ii) sCMMs visible, and iii) no distortion or impaired visibility of the core. Gold particles were counted in three zones: (1) CM, (2) sCMM zone and (3) core (see Supplementary Fig. S6). To select the different zones, images were loaded in ImageJ (Rasband, 1997-2018) and the CM was marked by using the “Segmented Line” tool. Next, membrane length was measured and then, the selection (marked by the segmented line) was enlarged by 10 nm by using the “Edit/Selection/Enlarge ...” function. The line limiting the enlarged selection was drawn in the image by using the “Draw” command and forms the outer border of the CM zone. Next, the selection was condensed by -20 nm using “Edit/Selection/Enlarge ...” function and again the line limiting this selection was drawn in the image thereby forming the inner border of the CM zone. The area limited by the current selection was measured using the “Measure ...” command, before the selection was further condensed by -40 nm to form the inner border of the sCMM zone which is at the same time the border of the core zone (Supplementary Fig. S6). The area of this final selection was also determined by using the “Measure …” command and represents the core zone area. The area of the sCMM zone was determined by calculating the difference between the area limited by the

inner border of the CM zone and the core zone area. Finally, gold particles were counted for each of the zones. Particles touching the lines of selection were attributed to a zone if they are located more than 50% within the respective zone. Particles which are located directly at the border were attributed to the CM zone and to the sCMM zone at the inner border of the sCMM zone. All data were collected in a spreadsheet, numbers for area and gold particles were summed for each data set and finally the particle density per membrane length (CM zone) or area (sCMM and core zone) was computed and related to a reference membrane length of 50 µm or area of 1 $µm^2$ respectively.

To assess differences between anti-SpoVAD and control labelling, the means and SDs of labelling densities produced by the three different control antibodies were calculated for each zone and background. The difference between anti-SpoVAD and mean control labelling densities was then expressed relative to the SD of the control labelling densities for each zone by calculating the difference between anti-SpoVAD and mean control labelling densities devided by the SD (Table 1).

Supplementary Figures

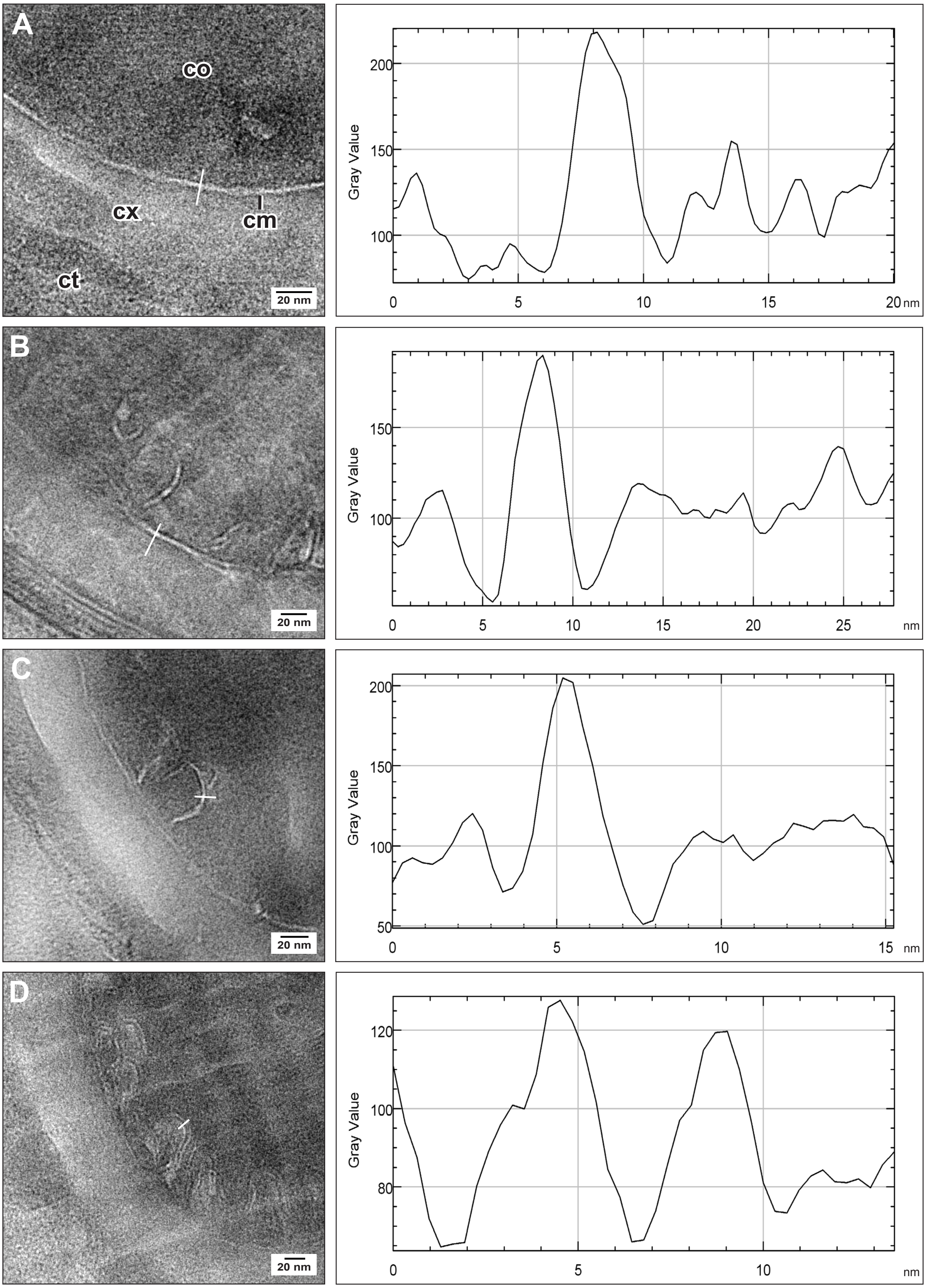

Supplementary Figure S1. Width of CM and sCMMs in dormant spores of *B. subtilis*. **Left side:** electron micrographs from cryo-electron microscopy of vitreous sections (CEMOVIS). **Right side:** line plots of gray values across CM or CMMs as indicated in the corresponding micrograph. (**A**), (**B**) CM. (**C**), (**D**) sCMMs with either a single dark-bright-dark (**C**) or a periodic dark-bright-dark-bright-dark contrast (**D**). The image in (**D**) is a magnified view of a spore cross-section already shown in Dittmann et al. (2015; Fig. 1A). Abbreviations: cm = core membrane, co = core, ct = coat, cx = cortex.

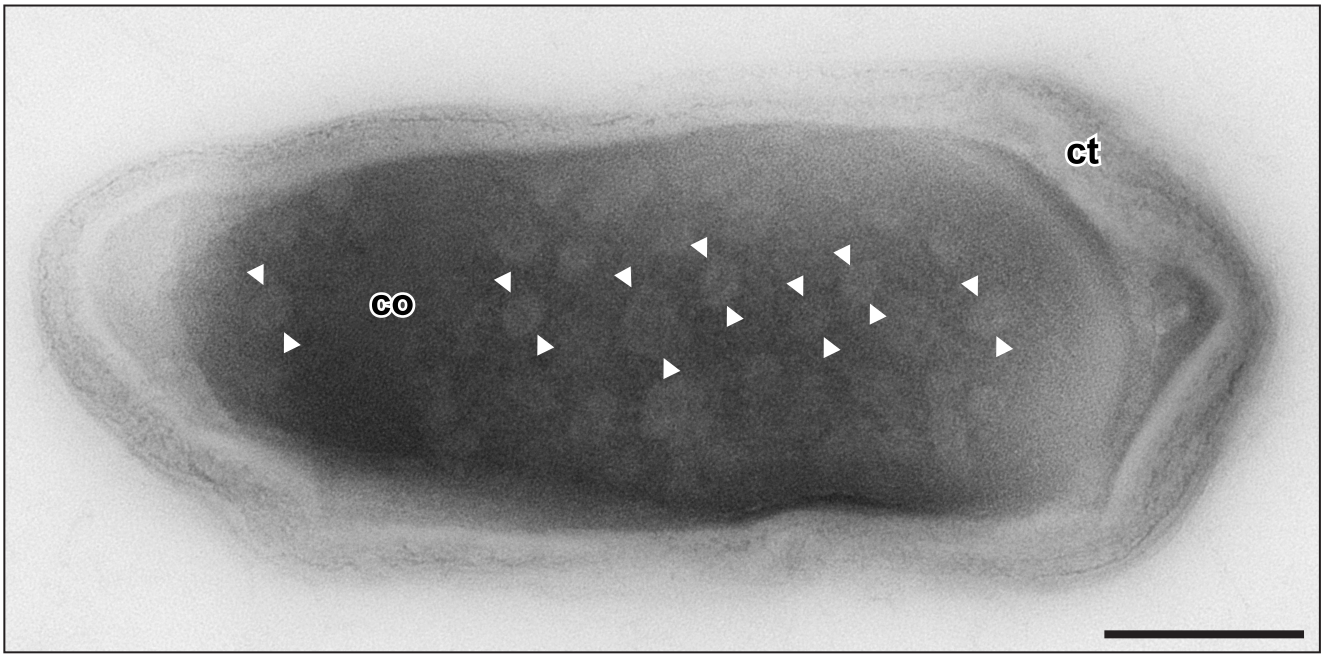

Supplementary Figure S2. Tangential section parallel to the long axis through a dormant *B. subtilis* spore visualized by conventional transmission electron microscopy. The core (co) shows numerous concentric structures (arrowheads), at lower electron density than the rest of the core, which are similar in size and distribution than the sCMMs as visualized in tangential sections by CEMOVIS (see Fig. 2C). Abbreviations: co = core, ct = coat, scale bar = 200 nm.

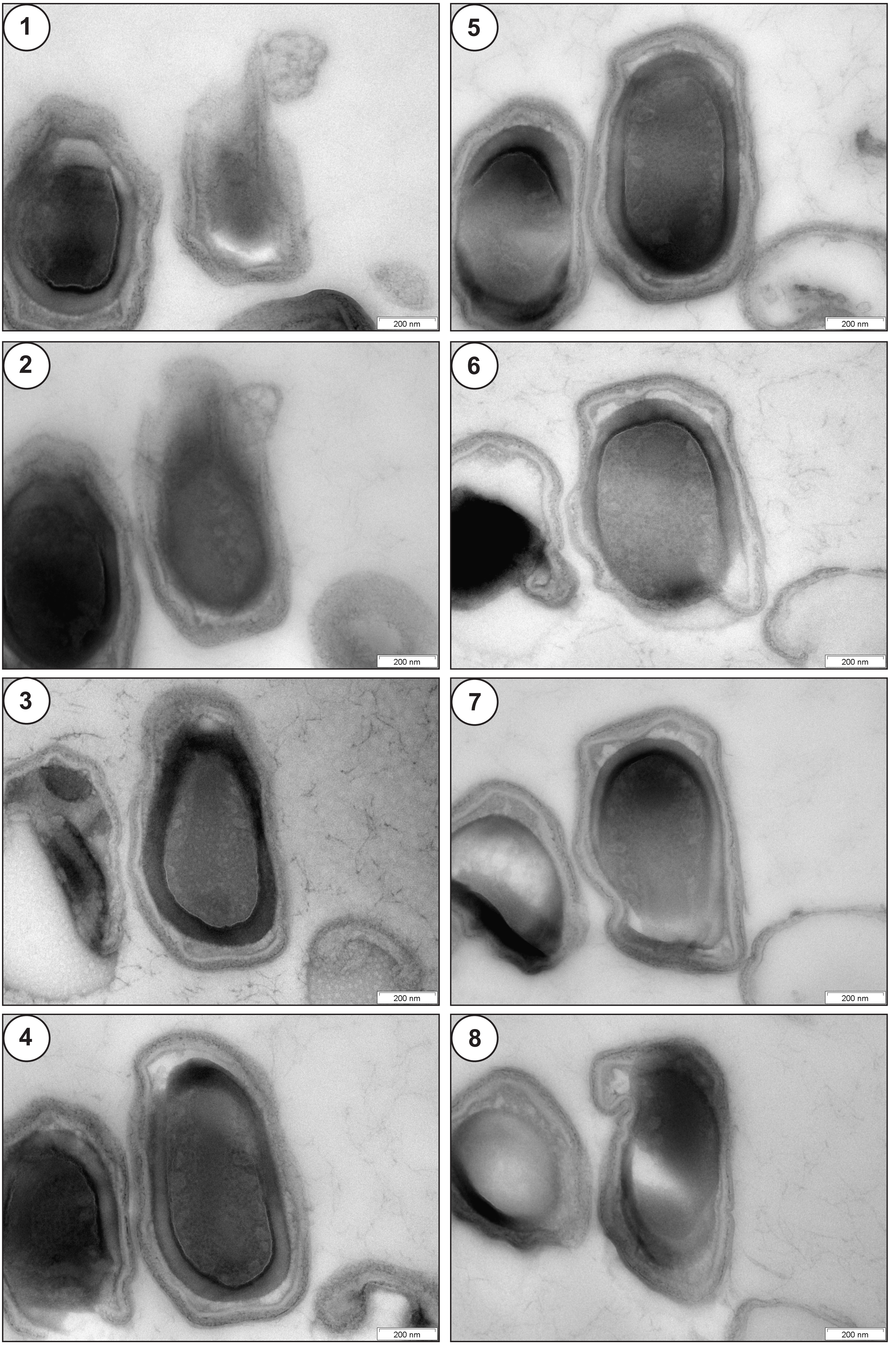
1
200 nm
2
200 nm
3
200 nm
4
200 nm
5
200 nm
6
200 nm
7
200 nm
8
200 nm

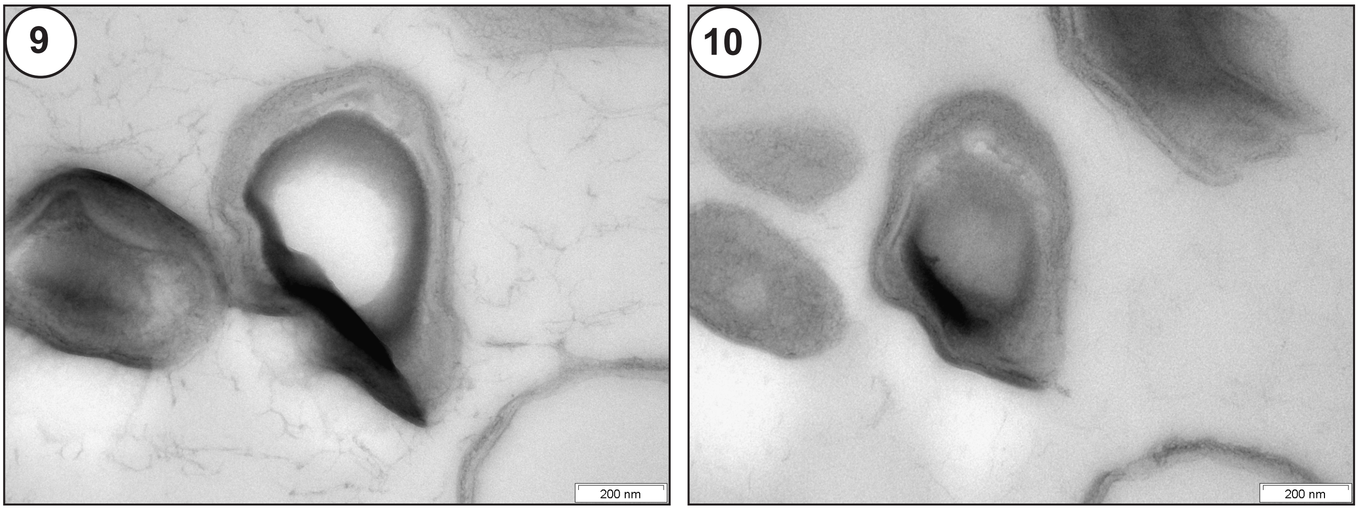

Supplementary Figure S3. Serial sections (No. 1 to 10) through the long axis of a dormant *B. subtilis* spore, visualized by conventional transmission electron microscopy. The sCMMs are localized below the CM without significant regional preference throughout the entire spore (see Fig. 4 for a description of structures).

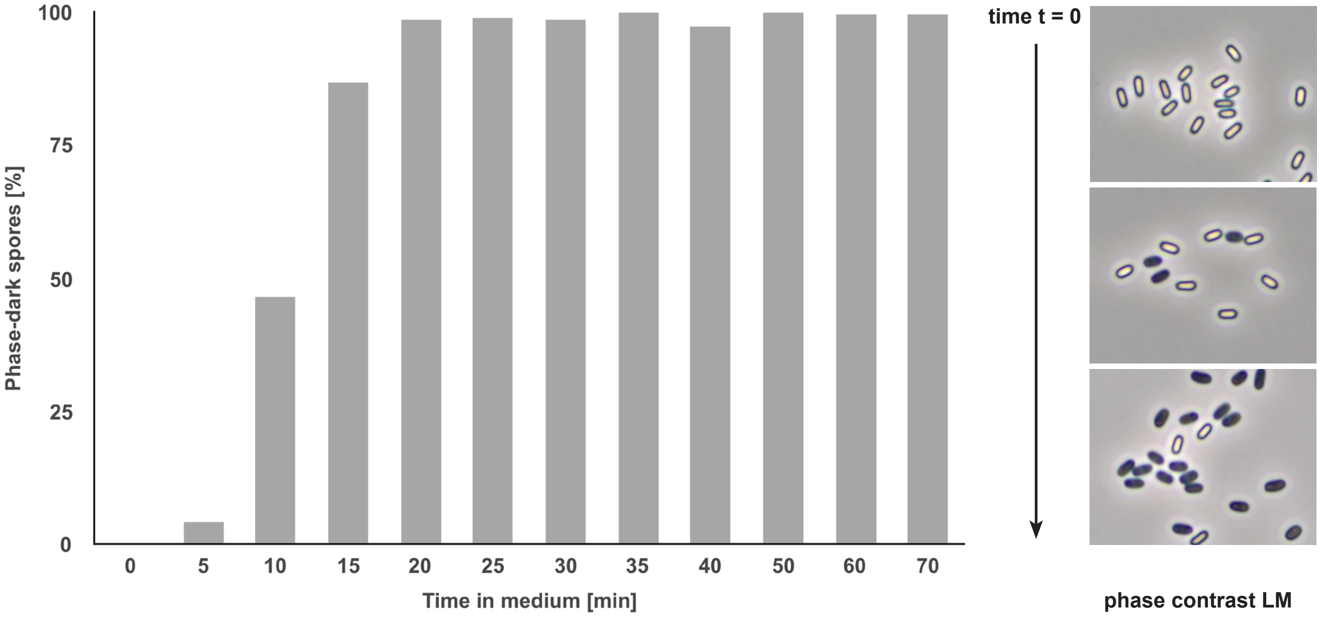

Supplementary Figure S4. Time course of germination of *B. subtilis* spores incubated in TSB medium at 37 °C on a shaker. The fraction of spores with a phase-dark core in phase-contrast light microscopy, which indicates at least release of most (70%; Kong et al. 2010) of the CaDPA stored in the core, is plotted against time of incubation in TSB medium. On the right side, representative micrographs show the spores in phase-contrast light microscopy at three different time points, starting with t = 0 min at the top. Note that this germination experiment has been also used in the study by Dittmann et al. (2015) to analyze the crystalline nucleoid of spores.

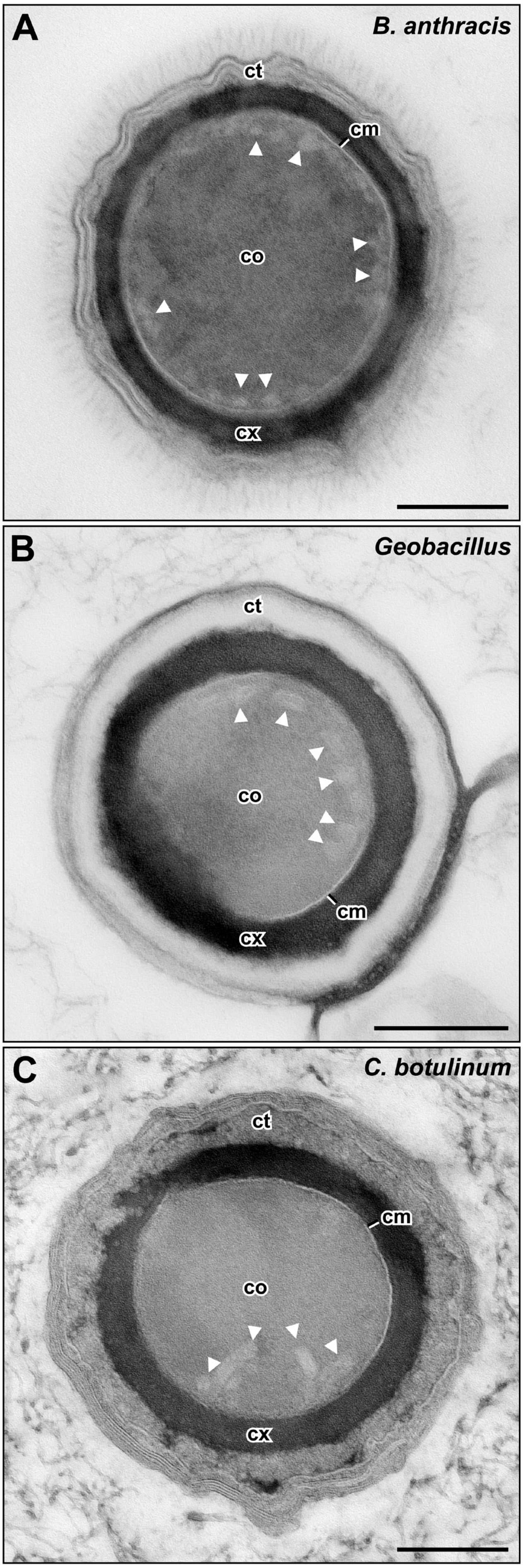

Supplementary Figure S5. sCMMs are present in spores of different species. The images show spo-re cross-sections of (**A**) *B. anthracis*, (**B**) *Geobacillus stearothermophilus* and (**C**) *C. botulinum*. The sCMMs (ar-rowheads) appear vesicular in *B. anthracis* and more tubular in *G. stearothermophilus* and *C. botulinum*. Abbreviations: cm = core membrane, co = core, ct = coat, cx = cortex, scale bars = 200 nm.

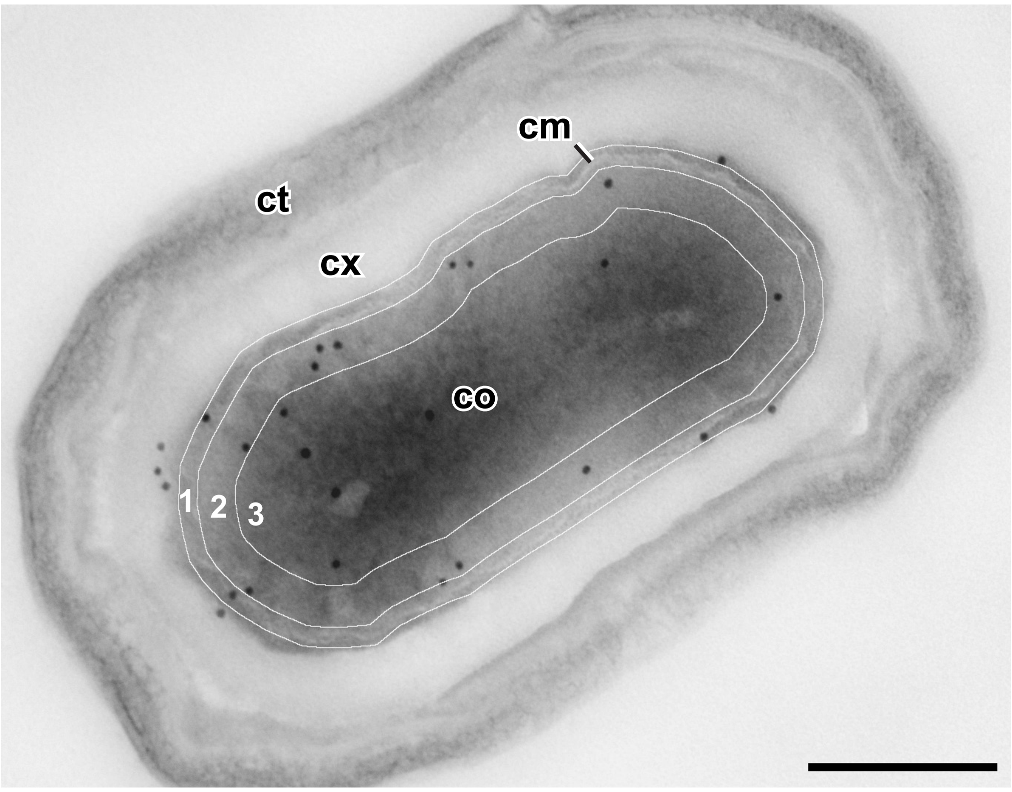

Supplementary Figure S6. Regional subdivision of the spore core into different zones for quantification of SpoVAD immunogold labelling of *B. subtilis* spore sections. 1 = CM, 20 nm wide; 2 = sCMM zone, 40 nm wide; 3 = core region below sCMMs. Abbreviations: cm = core membrane, co = core, ct = coat, cx = cortex, scale bar = 200 nm.

Supplementary Videos

Supplementary Video 1. Series of tomographic slices of a tomogram (SIRT reconstruction) from a thin plastic section through a dormant *Bacillus subtilis* spore.

Supplementary References

Dittmann, C., Han, H.-M., Grabenbauer, M. & Laue, M. Dormant *Bacillus* spores protect their DNA in crystalline nucleoids against environmental stress. *J. Struct. Biol.* 191, 156–164 (2015).

Griffiths, G. Fine-structure immunocytochemistry. (Springer, 1993).

Kong, L., Zhang. P., Setlow, P., Li, Y.Q. Characterization of bacterial spore germination using integrated phase contrast microscopy, Raman spectroscopy, and optical tweezers. *Anal. Chem.* 82, 3840-3847 (2010).

Rasband, W.S. ImageJ U. S. National Institutes of Health, Bethesda, Maryland, USA. https://imagej.nih.gov/ij/ (1997-2018).
